# Psychoeducation versus treatment as usual in diabetic patients with subthreshold depression: preliminary results of a randomized controlled trial

**DOI:** 10.1186/1745-6215-10-78

**Published:** 2009-08-26

**Authors:** Mirjana Pibernik-Okanovic, Drazen Begic, Dea Ajdukovic, Natasa Andrijasevic, Zeljko Metelko

**Affiliations:** 1 Vuk Vrhovac University Clinic, Dugi dol 4a, Zagreb, Croatia; 2 Rebro University Hospital, Kispaticeva 12, Zagreb, Croatia

## Abstract

**Background:**

Research on the effects of treating sub-threshold depression in persons with diabetes is scarce in spite of the findings indicating that this condition is highly prevalent in the diabetic population and may increase the risk of developing a subsequent major depression. This study was aimed at exploring the effects of a psycho-educational intervention on depression- and diabetes-related outcomes in patients with mild to moderate depressive symptoms.

**Methods:**

A randomized controlled study design with a one-year follow-up was used. Fifty patients with mild to moderate depressive symptoms (74% female, aged 57 ± 9 yrs, diabetes duration of 10 ± 8 yrs, BMI 31 ± 6 kg/m^2^, HbA1C 7.7% ± 1.4, 53% insulin treated) were randomly assigned to either an intervention or a control group. The intervention group underwent four psycho-educational sessions aimed at enabling self-management of depressive symptoms. The control group was informed about the screening results and depression treatment options while continuing diabetes treatment as usual. Both groups were contacted by phone in 2–3-month intervals, and re-assessed for depression after 6 and 12 months. Changes in depressive symptoms and glycaemic control were considered primary outcomes. Mann-Whitney U test and Friedman ANOVA were used to compare between- and within-group indicators at 6- and 12-month follow-ups.

**Results:**

Both the intervention and the control group reported a significant decrease in depressive symptoms as measured by the CES-D scale (Friedman ANOVA χ^2 ^= 10.8 p = .004 and χ^2 ^= 7.3 p = 0.03, respectively). The 6-month and 1-year indicators of glycaemic control as compared to baseline HbA1C values were also improved in both groups (χ^2 ^= 11.6 p = 0.003 and χ^2 ^= 17.1 p = 0.0002, respectively). Between-group differences in depressive symptoms and HbA1C values were not statistically significant either at 6- or at 12-month follow-up (all p > 0.05).

**Conclusion:**

Psycho-educational treatment appears to be beneficial in diabetic patients with mild to moderate depressive symptoms, but its effects are comparable with the non-specific support given to the subjects in the control group.

**Trial registration:**

Current Controlled Trials ISRCTN58745372

## Background

The prevalence of depression in diabetes is approximately twice as high as in the general population [1], implying a synergistic interaction between the two conditions that increases the risk of poor health outcomes [2].

In comparison with patients with diabetes alone, patients with both diabetes and depression have been shown to have poorer self-management (i.e. adherence to diet, exercise regimen and blood glucose monitoring) and significantly more lapses in refilling oral hypoglycaemic, lipid-lowering and antihypertensive prescriptions [3,4]. Depressed patients with diabetes are also significantly more likely to have cardiac risk factors such as smoking, obesity and sedentary lifestyle, compared to those with diabetes alone [5]. Depression is associated with an increased risk of metabolic dysregulation [6], micro- and macrovascular complications [7], and mortality [8].

Not only clinical depression but also its sub-threshold forms have been shown to have a profound influence on the affected patients' quality of life [9]. Defined as the presence of depressive symptoms that fall short of full diagnostic criteria for major depression or dysthymia, sub-threshold depression may be considered to be a part of a continuum of depressive disorders [10]. Judd et al. [11] conceptualized unipolar depression as presenting in different degrees of severity along a spectrum, with sub-threshold depression being the mildest form along the spectrum. It may represent a discrete category of its own but may also represent a prodromal, residual or interepisode symptomatic state in the course of major depression [12]. Data from the general population indicate that spontaneous improvement for this type of depression is low [13]. A systematic review of the literature on the prognosis of minor depression [14] showed that 16–62.3% individuals with sub-threshold depressive symptoms still have a minor depression after 5 months to 1 year of follow-up, suggesting that for many people this form of depression is chronic or recurrent. Sub-threshold depression has been found to increase the risk of subsequent major depression [15] and suicide [16]. Recent studies have uncovered some predictors of conversion from minor depression into its more severe clinical forms, chronic illness and medical burden being shown to be among them [17,18].

As research on treatments for sub-threshold depression in diabetic patients is scarce, data on their hypothetical effects on depression- and diabetes-related outcomes are inconclusive. There has been only one small randomized placebo-controlled pilot study of pharmacological treatment conducted in 15 mildly depressed women with type 2 diabetes [19], its results indicating beneficial treatment effects on insulin sensitivity. A small non-randomized study of the effects of a psycho-educational intervention on mood and glycaemic control in adults with diabetes and visual impairment [20] has shown positive effects on diabetes-related distress as measured by the Problem Areas in Diabetes scale, and on glycaemic control. The study has demonstrated significant positive correlation between glycaemic control and improvement in depression. Both of these studies have employed small sample sizes and study designs that do not allow reliable conclusions about the clinical benefits of treating sub-threshold depression in persons with diabetes.

The hypothesis of this study was that screening depressive symptoms in diabetic patients attending their regular medical check-ups, and including those with sub-threshold depression in a psychoeducational intervention accompanied by a structured follow-up, might have positive effects on depression- and diabetes-related outcomes as defined as improvement of depressive symptoms and glycaemic control. The study was expected to remedy methodological inadequacies inherent to previous studies in the field using a randomized controlled study design with a one-year follow-up. It was aimed at comparing the effects of the psycho-educational intervention in diabetic patients with mild to moderate depressive symptoms with those of standard diabetes care including screening for depression and a structured follow-up.

In this paper we present baseline and one-year follow-up data of 50 patients randomly assigned to the two groups.

## Methods

Diabetic patients attending their regular check-ups at the Vuk Vrhovac University Clinic for Diabetes, Endocrinology and Metabolic Diseases, a referral centre for the registration, treatment and follow-up of patients with diabetes in Croatia, were screened for depression by using the Patient Health Questionnaire (PHQ-9). Patients with scores of 10–14 points, which indicated mild to moderate depression [21], were the trial's target group. A history of poor literacy, mobility difficulties, visual impairment, drinking problems, co-morbid organic psychiatric disorder or psychosis was considered as the exclusion criteria.

The eligible patients were explained the purpose of the study and requested to give written consent to participate. Patients who were willing to be included were randomized to either the intervention or the control group by means of sequentially numbered sealed envelopes. Patients who refused to participate in the research received their usual diabetes care and were excluded from this study.

Participants in the intervention arm were included in a psycho-educational programme consisting of four interactive group sessions. The control subjects continued to receive standard diabetes care while being informed about the outcomes of the performed screening procedure, and about available treatment modalities. Both groups were followed for one year including re-assessments of depressive symptoms and glycaemic control at 6 and 12 months, and telephone calls in 2- to 3-month intervals to check on patients' actions in managing depression.

At baseline, the study participants were interviewed using a semi-structured interview inquiring about their psychological history (past psychological morbidity, method of treatment, course of symptoms, psychological morbidity in family members) and present psychosocial situation (family status, professional status, economic circumstances, recent stressful experiences, perceived social support).

Psychological questionnaires Center for Epidemiologic Studies Depression Scale [22], Problem Areas in Diabetes [23] scale, health-related quality of life questionnaire [24] and Summary of Diabetes Self-Care Activities [25] were applied to collect data about patients' emotional state and their experience in living with diabetes. The questionnaires were previously psychometrically evaluated in Croatian diabetic patients.

The Centre for Epidemiological Studies Depression (CES-D) scale is a 20-item, self-report scale that asks respondents to indicate the frequency of experiencing each of the 20 symptoms over the previous week. The instrument uses a 4-point response scale ranging from "rarely or none of the time" to "most or all of the time" with total scores ranging from 0 to 60. Higher scores indicate more severe depressive symptoms. A cut-point of ≥ 16 was considered indicative of elevated depressive symptoms.

The Problem Areas in Diabetes (PAID) questionnaire is a 20-item, self-report scale that asks respondents to rate how much of a problem they find each of the 20 diabetes-related issues. The answers are given on a 5-point scale ranging from 0 ("not a problem") to 4 ("serious problem"). The PAID scores are summed (with total scores ranging from 0 to 80) and transformed to a 0–100 scale with higher scores indicating more diabetes-related distress. Scores > 40 were considered indicative of high distress.

The short-form health survey (SF-12 v2) comprises self-assessments of general health, physical functioning, physical roles, bodily pain, vitality, social functioning, emotional roles and mental health. The raw scores for particular subscales are transformed to a 0–100 scale with higher scores indicating better health-related quality of life.

The Summary of Diabetes Self-Care Activities (SDSCA) is a brief self-report questionnaire of diabetes self-management that includes items assessing general diet, specific diet, exercise, blood glucose testing, foot care and smoking. The questionnaire asks the respondents about the frequency with which they performed self-care activities over the previous 7 days. Higher subscale scores indicate more regular performing of the self-care activities included.

Medical data were collected from the patients' medical records. HbA_1c _was determined by an automated immunoturbidimetric method using Bayer reagents (Tarrytown, Il, USA) on Olympus AU600 analyser (Olympus Optical Co., Tokyo, Japan) with a normal range from 3.5 to 5.7% [26].

### The intervention arm

#### Psycho-education on depression

The psycho-educational intervention comprised 4 interactive small group meetings, each lasting for 90 minutes, on the following topics:

• Symptoms of depression; interaction of depression and diabetes;

• Alleviating burden of depression through activities and problem solving;

• Associations between depression and cognitive processes – thoughts, beliefs and attitudes that induce and maintain depression; and

• Developing a personal plan for managing depression-related problems in the future.

The first two meetings were held within a week of each other, and the third and the fourth at two-week intervals. Patients were provided with a self-help manual for overcoming depressive difficulties based on the "Coping with depression" course by P.M. Lewinsohn [27,28]. The manual was given to the participating patients prior to the first session in order to make them familiar with the course contents and to facilitate reflecting their own experiences. The manual's structure aimed to stimulate introducing personal examples and making notes. The group sessions consisted of discussing particular topics rather than listening about them. A part of the manual was a workbook containing exercises to recognize depressive symptoms, become aware of daily activity patterns, plan more pleasurable activities, solve problems by using a four-step approach, and to recognize and modify cognitive patterns that contribute to maintenance of depression. The exercises were planned as homework. It included keeping mood and daily activities diary, planning daily activities to include more enjoyable ones, practicing a problem solving technique to manage personal problems the patients were faced with, and using the acquired knowledge to improve self-awareness, primarily with respect to automatic negative thoughts that worsen the depressive mood. The patients' experiences in going through the homework were discussed at the beginning of the subsequent session.

The manual was tested for comprehensibility and clarity in a group of diabetic patients (N = 8) with different demographic and disease-related characteristics. For the purpose of this study, the programme was partially modified and adjusted to diabetes-specific emotional problems.

### The control arm

#### Depression screening followed by standard diabetes treatment

The patients screened for depression demonstrating elevated result were given explanation of their result and were informed about available treatment options. The control participants were contacted by phone at the same intervals as the patients from the intervention group, and re-assessed for psychological variables after 6 and 12 months.

Sample size calculation was based on the absolute change in depressive symptoms as measured by the CES-D questionnaire from the run-in period to the 6- and 12-month follow-up assessments. To demonstrate a clinically meaningful difference in the CES-D scores with alpha = 0.05 and power of 90%, and assuming a common standard deviation of the CES-D scores of 8.4, 94 patients would be needed in each group.

These preliminary results were analysed using non-parametric statistics including medians and modes to describe measures of central tendencies and variability, Mann-Whitney U test to determine between-group differences at the three measurement points, and the Friedman ANOVA test to determine within-group differences in depression-related and metabolic outcomes.

### Ethical approval

The study was approved by the Vuk Vrhovac Clinic Ethics committee.

## Results

Demographic, disease-related and psychological characteristics of the intervention and the control group are presented in Table 1. The two groups were comparable with respect to age, gender, diabetes duration, body mass index, glycaemic control, depressive symptoms and diabetes-related emotional problems (all p > 0.05). Health-related quality of life was comparable in both groups with the exception of physical functioning which was shown to be slightly better in the intervention group (p = 0.02). Self-reported diabetes self-care was similar in both groups with respect to healthy eating, exercise, blood glucose self-monitoring and foot care (all p > 0.05). Adherence to diabetes-specific diet seemed to be greater in the control group (p = 0.03). The intervention group had a higher level of education than the control group (p = 0.01).

**Table 1:** Demographic, disease-related and psychological characteristics of the patients from the intervention and the control groups

	Intervention group Median (25–75)	Control group Median (25–75)	Z	p
**Age (yrs)**	55 (51–62)	58 (53–64)	-1.1	0.27
**Female (%)**	64	84		0.11
**Education (yrs)**	12 (8–14)	11 (8–11)	2.52	0.01**
**Diabetes duration (yrs)**	10 (3–14.5)	10.5 (4.5–13.5)	-0.51	0.61
**Body mass index (kg/m^2^)**	30.8 (26.7–35.8)	30.9 (27.9–30.4)	0.25	0.80
**HbA1C (%)**	7.5 (6.4–8.3)	7.7 (6.6–8.9)	-0.42	0.68
**PHQ-9 (score)**	13 (11–18)	13 (11–15)	0.23	0.81
**CES-D (score)**	26 (22–30)	24 (18–35)	1.03	0.31
**PAID (total score)**	51 (33–60)	45 (25–58)	0.91	0.36
**Negative emotions**	56 (33–67)	48 (21–63)	0.86	0.40
**Treatment**	33 (17–50)	33 (17–50)	-0,23	0.82
**Food**	42 (33–75)	58 (33–75)	-0.03	0.98
**Social support**	38 (13–63)	13 (0–50)	1.49	0.14
**SDSCA-diet**	4 (3–6)	4 (3–6)	0.07	0.94
**-specific diet**	3.5 (2–5.5)	5.5 (3.5–7)	-2.11	0.03*
**-exercise**	3.25 (1.5–5)	3 (1–3.5)	0.75	0.43
**-blood glucose monitoring**	7 (0.75–5.25)	6.5 (0.5–7)	0.69	0.49
**-foot care**	3.5 (0–7)	2.5 (0–7)	0.51	0.61
**SF – General health**	25 (0–25)	25 (0–50)	-0.06	0.95
**Physical functioning**	37.5 (25–50)	17 (0–37.5)	2.36	0.02*
**Role physical**	50 (25–62.75)	50 (31.25–62.5)	0.08	0.94
**Role emotional**	50 (50–50)	50 (32–62.5)	-0.30	0.77
**Bodily pain**	50 (25–62.5)	25 (25–75)	-0.05	0.96
**Mental health**	38 (25–50)	38 (25–50)	-0.45	0.65
**Vitality**	25 (25–50)	50 (25–50)	-1.22	0.22
**Social functioning**	37.5 (25–50)	50 (25–75)	-1.78	0.07

** significant at 99% confidence level

* significant at 95% confidence level

Between-group differences at the 6- and 12-month follow-up visits are presented in Table 2.

**Table 2:** Comparisons of depressive symptoms and glycaemic control between the intervention and the control group at 6 and 12 months

	Absolute change: Intervention versus Control group	U	z	p
**Depressive symptoms at 6 months (CES-D scores)**	**26 **(22–30) to **18 **(12.5–28.5) versus **24 **(18–35) to **20 **(16.5–27)	264.5	-0.49	0.63

**Depressive symptoms at 12 months (CES-D scores)**	**26 **(22–30) to **19**(11–26) versus **24 **(18–35) to **19 **(15–26)	295.5	-0.33	0.74

**Glycaemic control at 6 months (HbA_1_C)**	**7.5 **(6.4–8.3) to **7.3 **(6.3–7.6) versus **7.7 **(6.6–8.9) to **6.9 **(6.2–8.2)	279.0	0.19	0.86

**Glycaemic control at 12 months (HbA_1_C)**	**7.5 **(6.4–8.3) to **7.0 **(6.0–7.6) versus **7.7 **(6.6–8.9) to **7.0 **(5.9–7.9)	293.5	-0.13	0.89

Both the intervention and the control group reported less depressive symptoms at the follow-up assessments and had better glycaemic control as compared to baseline indicators. The between-group differences were not statistically significant either at 6- or at 12-month follow-ups.

**Figure 1:**
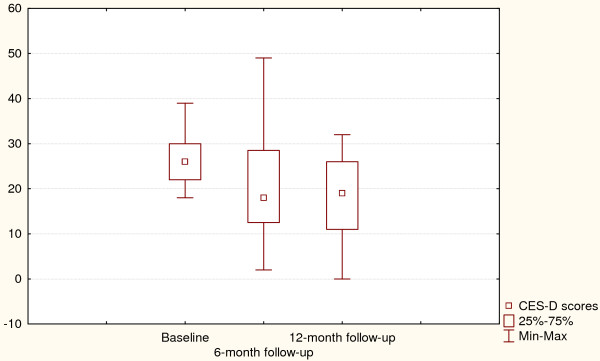
**Depressive symptoms at baseline and after 6- and 12-month follow-up (Intervention arm)**. Χ^2 ^= 10.8, p = 0.004, Coefficient of concordance = 0.27, Average rank correlation = 0.23.

**Figure 2:**
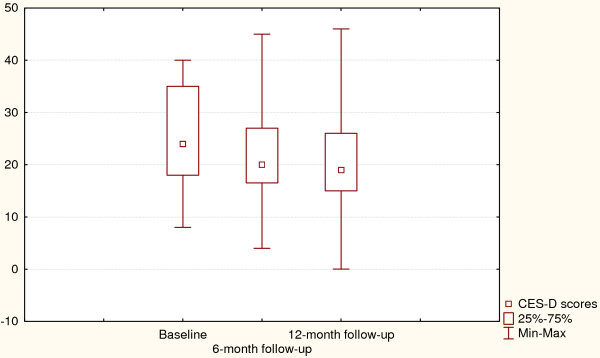
**Depressive symptoms at baseline and after 6- and 12-month follow-up (Control arm)**. Χ^2 ^= 7.3, p = 0.03, Coefficient of concordance = 0.19, Average rank correlation = 0.15.

Changes in depressive symptoms and HbA1C values for the intervention group are presented in Figures 1 and 2. Friedman ANOVA indicated that individuals treated with psycho-educational intervention reported improved depressive symptoms at the 6-month assessment and remained so after 12 months (p = 0.004). The same trend could be observed for HbA1C values which were significantly lower at the follow-up assessments, showing an average decrease of 0.5% (p = 0.0003).

**Figure 3:**
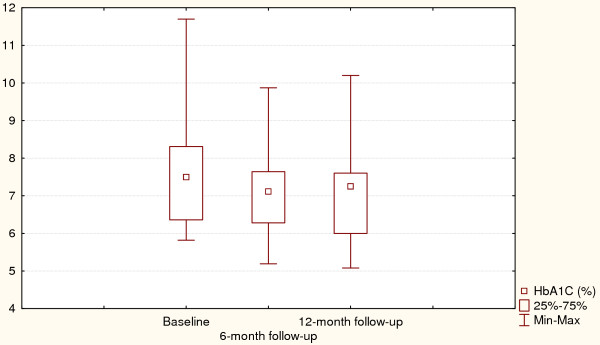
**Glycaemic control at baseline and after 6- and 12-month follow-up (Intervention arm)**. Χ^2 ^= 11.6, p = 0.003, Coefficient of concordance = 0.34, Average rank correlation = 0.30.

**Figure 4:**
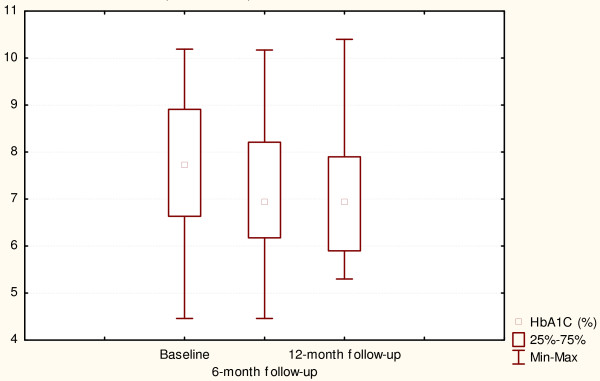
**Glycaemic control at baseline and after 6- and 12-month follow-up (Control arm)**. Χ^2 ^= 17.1, p = 0.002, Coefficient of concordance = 0.45, Average rank correlation = 0.42.

Changes in depression-related outcomes and glycaemic control for the control group are presented in Figures 3 and 4. Like the intervention group, the control subjects improved their depressive symptoms and HbA1C at 6- and 12-month follow-up assessments (p = 0.03 and p = 0.0002 respectively).

## Discussion

The preliminary data on the effects of the psycho-educational intervention in patients with mild to moderate depressive symptoms do not support its effectiveness in comparison with the non-specific support given to the control patients. A comparable improvement in depressive symptoms observed in the patients who were included in the psycho-educational group sessions, and in those who were only screened for depression and then followed for one year might suggest that treating sub-threshold forms of depression does not demonstrate a clear clinical utility. Such a conclusion might be additionally supported by the finding that both the intervention and the control participants demonstrated a similar improvement in glycaemic control at 6- and 12-month follow-up assessments. These findings suggest that the patients included in the study benefited in terms of improved mood and glycaemic control regardless of the study arm.

There are two hypothetical explanations of the results obtained. The first one concerns the structure of the control arm. Although defined as "diabetes treatment as usual" it actually implied a more supportive approach than diabetic patients usually receive within their standard care. Screening for depression and discussing the results with the patients may be considered a kind of an intervention as well. As shown by Pouwer et al. [29], monitoring and discussing psychological well-being as part of routine diabetes outpatient care had favourable effects on the patients' mood. Besides monitoring, the control participants in our trial received several telephone calls during the follow-up period, and were invited for depression reassessment after 6 and 12 months. This could have been experienced as an additional support possibly affecting the obtained results. Qualitative data on patients' experiences with participating in the trial collected at the end of the follow-up period support the hypothesis on the beneficial effect of monitoring patients' mood within standard diabetes care.

The second explanation of the obtained results concerns the intervention format and content. A short intervention used in the trial relied on cognitive-behavioural principles. It aimed to stimulate patients' activation and improve their capabilities to actively participate in solving their internal and external problems. However, some individuals found participation in group sessions and exercises difficult. Possibly due to their demographic characteristics (middle age, relatively low level of education, limited objective resources) they perceived engagement in psychological processes they had not previously practiced as difficult. Their ambivalence towards experimenting with new cognitive patterns might be even increased by the fact that, although agreeing to the intervention, they actually would not choose it if it were not recommended. Being asked about subjectively perceived benefits of the intervention at the end of the follow-up, some patients pointed out the new skills they learned, but the majority found the experienced support to be most helpful.

Qualitative data collected from the intervention and the control subjects allow a hypothesis that the two study arms had at least one common component, described by the patients as a sense of being supported and cared for, and that this component itself seems to be helpful in addressing sub-threshold depression in patients with diabetes.

Another relevant finding obtained in the study was that the intervention and the control groups comparably improved HbA1C values after 6- and 12-month follow-up periods indicating an inverse relation between depressive symptoms and glycaemic control. At present, the relationship between depressive symptoms and glycaemic control is still not fully understood. Some studies have proved an undesirable association between depressive symptoms and metabolic indicators [6] but others did not confirm such an association [30,31]. Effects of treating depressed diabetic individuals on their glycaemic control are also a matter of debate, with controversial reports on the association between metabolic improvement and reduction in depressive symptoms [32,33].

Our preliminary data suggest that focusing on patients' emotional state either in the form of a psycho-educational intervention or in the form of monitoring and following-up its further development, has positive effects on glycaemic control.

A limitation of this preliminary report is its smaller sample size than indicated by the power analysis. However, the preliminary data trend and the qualitative indicators of the patients' benefits gained from participating in the trial make these findings worth reporting.

Although slightly different with respect to education, self-reported adherence to diabetes-specific diet and self-reported physical functioning, the two groups could be considered basically comparable regarding disease-related and psychological variables. In accordance with the literature [34], depressive symptoms in our study participants frequently co-occurred with diabetes-related distress suggesting that focus should be equally on monitoring depressive symptoms and monitoring emotional distress caused by diabetes.

Further research relying on bigger sample sizes is needed to determine whether a psycho-educational intervention may be more efficient than monitoring and following well-being in patients with sub-threshold depression. Inquiring into patients' beliefs about the necessity of treating sub-threshold depressive symptoms, and value-weighted preferences regarding the treatment form may be helpful in determining which patients benefit the most.

## Conclusion

Preliminary data of the randomized controlled trial aimed at comparing the effects of a psycho-educational intervention in patients with mild to moderate depressive symptoms with screening for depression accompanied by a structured follow-up showed comparable improvements in depression- and disease-related variables in both study arms. The findings suggest that monitoring patients' well-being within diabetes check-ups, and following those with mild to moderate depressive symptoms could be sufficient at these early stages of depression development.

## Competing interests

The authors declare that they have no competing interests.

## Authors' contributions

MPO is the principal researcher who developed the original idea and design of the study, and drafted the manuscript. ZM participated in planning of the intervention evaluated in the paper. DB, DA and NA participated in data collection and interpretation, as well as in carrying out the study. All authors have read and corrected draft versions, and approved the final version.
